# Calf Circumference as a Novel Tool for Risk of Disability of the Elderly Population

**DOI:** 10.1038/s41598-017-16347-9

**Published:** 2017-11-27

**Authors:** Yu-Shan Sun, Tung-Wei Kao, Yaw-Wen Chang, Wen-Hui Fang, Chung-Ching Wang, Li-Wei Wu, Hui-Fang Yang, Fang-Yih Liaw, Wei-Liang Chen

**Affiliations:** 1 Division of Family Medicine, Department of Family and Community Medicine, Tri-Service General Hospital; and School of Medicine, National Defense Medical Center, Taipei, Taiwan; 2 Division of Geriatric Medicine, Department of Family and Community Medicine, Tri-Service General Hospital; and School of Medicine, National Defense Medical Center, Taipei, Taiwan; 3 Graduate Institute of Medical Sciences, National Defense Medical Center, Taipei, Taiwan

## Abstract

Disability became increasingly common with age, and crude rates of disability were rising around the globe. The aim of this study was to investigate the association between calf circumference (CC) and disability in the U.S. elderly population. From the 1999–2006 National Health and Nutrition Examination Survey, a total of 4,245 participants with an age range of 60–84 years were included. Disability was defined as the total number of difficulties within the following 5 major domains of disability, such as activities of daily living (ADL), instrumental ADL, general physical activities, lower extremity mobility, and leisure and social activities. The association between CC and disability was investigated through the regression model adjusted for multiple covariates. According to the fully adjusted model regarding disability, the β coefficients for each quartile of increasing CC were −0.041 for quartile 2 (P = 0.096), −0.060 for quartile 3 (P = 0.027), and −0.073 for quartile 4 (P = 0.026) respectively, compared with lowest quartile. There was a negative association between CC and disability among the elderly population. Calf circumference may be a novel risk assessment for disability of elderly people.

## Introduction

Disability is defined as a limitation in the ability to carry out basic functional activities and becomes increasingly common with age. Crude rates of disability are rising around the globe^1^; nearly one in four United States Medicare beneficiaries report at least one health-related disability. Disability has a large impact on the quality of life of an individual and the caregiver; furthermore, disability is an independent risk factor for increased health care costs^2^.

Disability in the elderly population has been known to be associated with multiple chronic conditions, such as chronic inflammatory conditions (diabetes, metabolic syndrome)^3^, affective disorders (depression)^4^, and neurologic conditions (dementia, stroke, Parkinsonism)^5^. Despite the disease condition, the evidence from several recent studies has demonstrated an association between disability and the muscle mass and muscle strength of elderly people^6–8^. In addition, several studies have shown an association between a pre-frail and frail status and an increased risk of disability in the elderly population^9,10^.

The measurement of anthropometric parameters is a non-invasive, cost-effective way to evaluate the health and nutrition status of elderly people. According to the World Health Organization international database, aging and associated diseases significantly influence anthropometric data^11^. Reports of a previous study show a greater proportion of frailty in older people with a large waist circumference (WC); moreover, an association was found between high body mass index (BMI) and increased functional disability and the presence of comorbidities and coexisting factors of frailty^12–14^. In a Swedish study including 3,360 participants, subjects with activities of daily living (ADL)-dependence had significantly larger BMI, waist, and lower triceps- and subscapular-skinfold thickness^15^. The findings may indicate that inflammation and insulin resistance are the possible mechanism of frailty and disability.

Calf circumference (CC) is an anthropometric parameter that is closely related to whole body muscle mass^16^ and is known to be associated with the nutrition status of the elderly population. In a Japanese study including 526 participants, CC was positively correlated with appendicular skeletal muscle mass and skeletal muscle index, and was associated with the diagnosis of sarcopenia^17^. Furthermore, weight-adjusted CC showed negatively correlated with insulin resistance indices, such as H-IR and HOMA-IR^18^. A Korean study also found that lower body circumferences has associate with metabolic syndrome and arterial stiffness^19^. The findings indicate that CC, related to insulin resistant, may have associated with disability of elderly population. However, the role of CC in the evaluation of elderly disability remains unknown. Therefore, we collected data from the 1999 to 2006 National Health and Nutrition Examination Survey (NHANES) to evaluate the association between CC and disability of elderly people in the U.S.

## Results

### Characteristics of the study population and association of disability and anthropometric parameter

A total of 41,474 participants were included in the NHANES dataset from 1999 to 2006. We included a total of 5,816 participants between the age of 60 and 84 years. We further excluded 1,571 persons with missing data from a laboratory or clinical examination (N = 884), and disability questionnaire screening (N = 687). Finally, 4,245 participants were included in our study. We examined the association of disability and anthropometric parameter by a regression model (Table 1), which showed CC has negative association with disability in elderly people (p < 0.05).

**Table 1:** Association between disability and anthropometric parameters.

Anthropo-metric parameters	Model^a^ 1 β^b^ (95% CI)	*P* Value	Model^a^ 2 β^b^ (95% CI)	*P* Value	Model^a^ 3 β^b^ (95% CI)	*P* Value
AC (cm)	−0.072 (−0.102, 0.009)	0.101	−0.073 (−0.103, 0.009)	0.350	−0.066 (−0.096, 0.011)	0.123
WC (cm)	0.189 (0.020, 0.056)	<0.001	0.186 (0.019, 0.056)	0.002	0.186 (0.020, 0.055)	<0.001
TC (cm)	−0.019 (−0.045, 0.029)	0.660	−0.011 (−0.042, 0.033)	0.474	0.013 (−0.030, 0.042)	0.752
CC (cm)	−0.118 (−0.134, −0.031)	0.002	−0.117 (−0.134, −0.030)	0.002	−0.077 (−0.104, −0.004)	0.035

^a^Adjusted covariates:

Model 1 = age, sex, race/ethnicity, BMI.

Model 2 = Model 1 + serum fasting glucose, serum cholesterol, AST, HDL.

Model 3 = Model 2 + history of congestive heart failure, coronary heart disease, stroke, Hypertension, Diabetes, current smoke, experience of memory problem.

^b^β coefficients was interpreted as change of disability for each increase in different anthropometric parameters.

Abbreviation:

AC, arm circumference; WC, waist circumference; TC, thigh circumference; CC, calf circumference; BMI, body mass index; AST, aspartate aminotransferase; HDL, High-density lipoprotein.

Demographic, biochemical and clinical characteristics of the study population stratified according to CC quartiles are shown in Table 2. The mean age of all participants was 70.21 ± 7.03 years, and 50.3% of participants were male. Compared to subjects in the lower quartiles, participants in the higher CC quartiles tended to have a higher BMI, lower HDL level, higher frequency of ever being diagnosed with hypertension, and history of memory problem (Table 2).

**Table 2:** Characteristics of study participants of quartiles of calf circumference.

Characteristics of Study Participants	Quartiles of Calf Circumference					
	Q1 (<34.8) (n = 1079)	Q2 (34.8 to <37.2) (n = 1087)	Q3 (37.2 to <39.9) (n = 1037)	Q4 (>39.9) (n = 1042)	Total (n = 4245)	*P* Value
Continuous variables^a^						
Age (years)	71.91 (7.24)	70.98 (7.04)	69.74 (6.94)	68.13 (6.30)	70.21 (7.03)	<0.001
BMI (kg/m^2^)	23.89 (3.38)	26.72 (3.26)	29.38 (3.66)	33.97 (5.34)	28.43 (5.44)	<0.001
Serum FG (mg/dL)	107.82 (44.69)	106.11 (39.47)	105.38 (34.88)	108.34 (35.02)	106.91 (38.76)	0.262
Serum TC (mg/dL)	208.72 (45.30)	207.93 (42.61)	204.09 (41.30)	204.28 (41.23)	206.28 (42.69)	0.019
AST (U/L)	26.11 (51.91)	23.91 (7.36)	23.93 (8.63)	24.09 (7.80)	24.51 (26.93)	0.178
Serum HDL (mg/dL)	57.63 (17.46)	54.54 (16.48)	52.34 (15.89)	51.44 (14.32)	54.01 (16.26)	<0.001
Categorical variables^b^ (%)						
Male	39.4	50.8	56.7	54.8	50.3	<0.001
Non-Hispanic white	53.4	58.8	62.8	64.8	59.9	<0.001
ADL impairment	14.9	12.1	13.3	17.3	14.4	0.004
IADL impairment	20.8	13.4	16.5	18.4	17.3	<0.001
LSA impairment	14.0	8.9	11.0	15.6	12.3	<0.001
LEM impairment	18.0	15.6	17.5	22.2	18.2	0.003
GPA impairment	47.9	39.3	43.6	51.4	45.5	<0.001
CHF	7.0	5.0	6.6	7.6	6.5	0.093
CHD	16.2	15.9	17.3	17.2	16.6	0.763
Stroke	8.3	6.1	6.7	5.5	6.6	0.058
Hypertension	69.0	65.8	70.1	74.9	69.9	<0.001
Diabetes	20.7	18.3	19.1	21.1	19.8	0.336
Current Smoker	27.9	15.8	14.3	12.0	17.7	<0.001
Had memory problem	16.7	9.9	10.2	8.8	11.4	<0.001

BMI, body mass index; Serum FG, serum fasting glucose; Serum TC, serum total cholesterol; AST, aspartate aminotransferase; HDL, High-density lipoprotein; ADL, Activity of daily living; IADL, instrumental Activities of daily living; GPA, general physical activities; LME, lower extremity mobility; LSA, leisure and social activities, CHF, Congestive heart failure; CHD, Coronary heart disease.

^a^Values were expressed as mean (standard deviation).

^b^Values in the categorical variables were expressed as %.

### Association of disability and calf circumference

**Figure 1:**
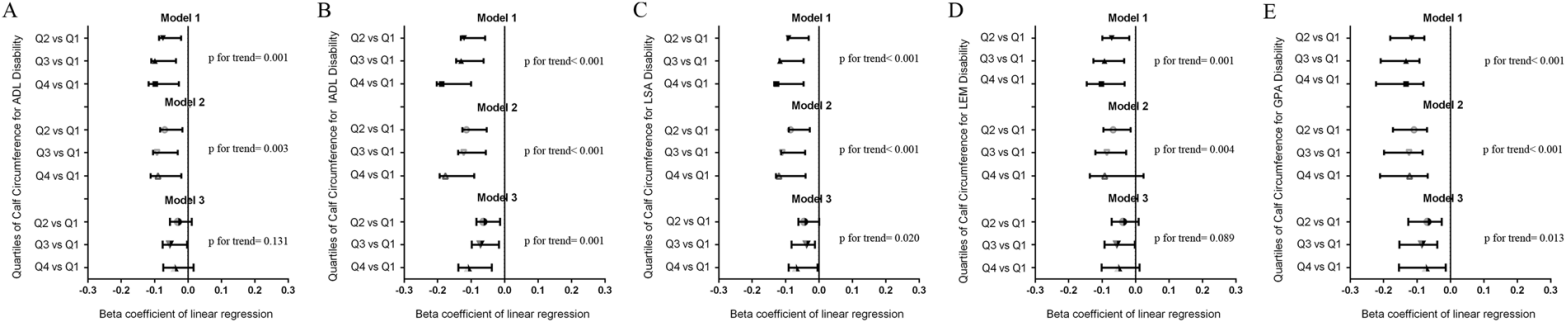
(**A**) Association between ADL disability and quartiles of calf circumference. (**B**) Association between IADL disability and quartiles of calf circumference. (**C**) Association between LSA disability and quartiles of calf circumference. (**D**) Association between LEM disability and quartiles of calf circumference. (**E**) Association between GPA disability and quartiles of calf circumference.

The results for the association between disability and CC quartile are presented in Table 3. According to the quartile-based analysis, the β coefficients of predicted total disability compared between the CC quartiles in the adjusted model were −0.041 (P = 0.096), −0.060(P = 0.027), and −0.073 (P = 0.026) (p = 0.022 for trend). The associations between the five disability domains and CC quartiles are presented in Fig. 1. In the fully adjusted model, IADL disability and GPA disability were significantly associated with CC in quartile, the β coefficients of predicted total disability were −0.063 (P = 0.007), −0.072 (P = 0.005), and −0.109 (P = 0.001), (P = 0.001 for trend) in IADL and −0.068 (P = 0.003), −0.085 (P = 0.001), and −0.073 (P = 0.019) (p = 0.013 for trend) in GPA (Fig. 1).

**Table 3:** Association between the quartiles of calf circumference and disability.

Models^a^	Calf Circumference Quartiles	β^b^ (95% CI)	*P* Value	*P* for Trend
Model 1	Q2 v.s. Q1	−0.086 (−0.815, −0.224)	0.001<	<0.001
	Q3 v.s. Q1	−0.108 (−1.000, −0.330)	0.001<	
	Q4 v.s. Q1	−0.138 (−1.279, −0.452)	0.001	
Model 2	Q2 v.s. Q1	−0.082 (−0.787, −0.195)	0.001<	<0.001
	Q3 v.s. Q1	−0.102 (−0.964, −0.292)	0.001<	
	Q4 v.s. Q1	−0.130 (−1.229, −0.397)	0.001	
Model 3	Q2 v.s. Q1	−0.041 (−0.532, 0.043)	0.096	0.022
	Q3 v.s. Q1	−0.060 (−0.694, −0.041)	0.027	
	Q4 v.s. Q1	−0.073 (−0.864, −0.055)	0.026	

^a^Adjusted covariates:

Model 1 = age, sex, race/ethnicity, BMI.

Model 2 = Model 1 + serum fasting glucose, serum cholesterol, AST, HDL.

Model 3 = Model 2 + history of congestive heart failure, coronary heart disease, stroke, Hypertension, Diabetes, current smoke, experience of memory problem.

^b^β coefficients was interpreted as change of disability for each increase in different anthropometric parameters.

Abbreviation:

BMI, body mass index; AST, aspartate aminotransferase; HDL, High-density lipoprotein.

## Discussion

To our knowledge, this is the first study to demonstrate CC as a useful parameter related to the risk of disability in the elderly population. By analyzing the representative sample of the U.S. population, we determined if there is an association between disability and the following anthropometric parameters: waist circumference, arm circumference, thigh circumference and calf circumference (CC). Of the four anthropometric parameters, only CC had a significant inverse association with disability of elderly individuals between the age of 60–84 years. In a previous study, CC was found to have a positive association with functional performance and opposite association with risk of mortality and frailty index in an elderly population^20^. Low CC was found associated with slow walking speed in South America elderly population^21^. In Taiwan Longitudinal Survey on Aging study including 2521 participants, compared to BMI, CC has better ability to predict emerging care-need in older Taiwanese^22^.

CC, which can reflect changes in fat-free mass, was a sensitive measure of muscle mass and peripheral subcutaneous fat in elderly people^11^. In our study, we found a significant inverse association between CC and disability in elderly individuals. There are several plausible explanations for our finding. First, there was a close relationship between CC and whole body muscle mass^16^. Muscle mass is a metabolic tissue and endocrine organ, and the construction of muscle mass releases several endocrines called myokines^23,24^. Some of the myokines may be involved in mediating the inflammation process, and may lead the anti-inflammation effect^25,26^. The chronic inflammation status was highly associated with elderly disability and mortality. In a previous study, high serum levels of interleukin-6 (IL-6), tumor necrosis factor-α (TNF-α) and C-reactive protein (CRP) were found to increase the incidence of limited mobility in elderly people^27^. High levels of IL-6 and CRP were also found to be significantly associated with poor physical performance in older populations in the InCHIANTI Study^28^. In addition, reduced relative skeletal muscle mass is known to be significantly and independently associated with functional impairment and disability^29^. Collectively, the anti-inflammation effect accompanied by increased muscle mass may be one mechanism for the association of CC with disability.

In addition to the anti-inflammatory effect, myokines also play an important role in the insulin-signaling pathway^30–32^. Both insulin and insulin-like growth factor-1 (IGF-1) may promote glucose transport in muscle and have growth-promoting activities in muscle^33^. Emerging evidence has addressed that low muscle density is associated with low insulin sensitivity^34^, and low arm and leg muscle mass to body weight ratio was associated with a higher prevalence of metabolic syndrome^19,35^. Furthermore, some animal studies have revealed that the loss of the IGF-1 sensitizing actions in skeletal muscle leads to the development of insulin resistance^36,37^. According to accumulating studies, insulin resistance is associated with muscle strength, power and muscle mass in elderly people^38,39^.

Lower plasma IGF-1 levels were found to be associated with poorer muscular strength, walking speed, mobility tasks, physical performance, and all-cause mortality in the elderly population^40,41^. Metabolic syndrome, representing the combination of central obesity and insulin resistance, was found to have an association with elderly disability^3^. In recent Japanese study showed that weight-adjusted CC negatively correlated with blood pressure; Triglyceride, fasting plasma glucose, HbA1c, and insulin resistance indices, such as hepatic insulin resistance (H-IR) index and homeostasis model assessment of insulin resistance (HOMA-IR) index^18^. Accordingly, insulin resistance, which is related to reduced muscle mass, may be one mechanism for the negative association between CC and disability.

There are several limitations of this study. First, the study was a retrospective observational analysis of the database, which was collected at a single time point, rather than a long-term repeated observation; the direction of association may not be determined through a retrospective observational analysis. Second, we used the 1999–2006 NHANS data due to only the data in those years included both functional disability questionnaire results and anthropometric parameters result. There may be few demographic changes in U.S population between those years and present day. However, based on the Current Population Reports of U.S Census Bureau^42^, U.S aging population aged more than 65 years was approximate 14.8% in 2015. The population aged 65 years and over from 1999–2006 NHANES dataset was 14.02%. Our datasets, which included 4,245 U.S elderly participants, may still be representative of the current U.S population of community-dwelling older adults. Third, we only used the single self-reported results to determine disability, which may have led to over-reporting participants; the results may have been affected by recall bias or misclassification and may not represent real disability conditions among the participants. Participants may have been disabled at the time of the interview and recovered soon after. Finally, despite adjusting for potentially confounding factors, there may have been residual effects from unadjusted confounding factors of the association between anthropometric parameters and disability.

The aim of this study was to define the relationship between CC and the risk of disability in U.S. elderly individuals aged 60–84 years. Calf circumference is an important anthropometric parameter that should be considered in geriatric assessments, especially for disabled elderly populations. Further research is necessary to determine whether or not CC may be used to predict disability in the elderly population.

## Methods

### Ethics statement

The NHANES study protocol was approved by the NCHS Institutional Review Board (IRB). Before data collection procedures and NHANES health examinations, all informed consents had been obtained from the eligible subjects.

### Study populations

NHANES is a cross-sectional study that was conducted by the Centers for Disease Control and Prevention (CDC), National Center for Health Statistics (NCHS). The aim of the study was to assess the health and nutritional information of non-institutionalized U.S. civilians. The survey included an extensive household interview (information on age, sex, race, and medical history) and a subsequent physical examination at a specially equipped Mobile Examination Center (MEC). We included all participants above the age of 60 years from the 1999–2006 cohort, which included body measurement data and information on functional status. We excluded participants with missing information, or other demographic or related covariates.

### Anthropometric parameter

The data from the 1999–2006 NHANES and body measurement assessments were collected in MEC by well-trained health technicians. Arm and leg measurements were taken on the right side of the body. If a participant had any condition such that measurements could not be taken on the right side of the body, such as an amputation or medical appliance, the examiner took measurements on the left side. Arm circumference was at the middle point of the upper arm, perpendicular to the long axis of the upper arm, while the participant standing with shoulders relaxed and arm hanging loosely. Waist circumference was measured at the high point of the iliac crest at minimal respiration to the nearest 0.1 cm. Thigh circumference was measured at the middle point of the thigh, perpendicular to the long axis of the thigh, while the participant was standing with most of the weight on the left leg and the right leg forward, knee slightly flexed, and soles of both feet flat on the floor. CC was measured at a plane perpendicular to the long axis of the calf while the participant was sitting on chair with right foot flat on the floor.

### Definition and data collection of disability

To evaluate functional status, participants were asked 19 questions associated with functional mobility and transfers, household productivity, manipulation of surroundings, and social integration. The 19 questions were divided into the following 5 domains: (1) activities of daily living (ADL) (dressing, eating, getting out of bed, and walking), (2) instrumental activities of daily living (IADL) (preparing meals, managing money, and doing house work), (3) leisure and social activities (LSA) (going out to watch movies, attending social events, and in-home leisure activities), (4) general physical activities (GPA) (standing, lifting, bending, sitting, stooping, grasping, and reaching), and (5) lower extremity mobility (LEM) (walking 1320 feet and walking up 10 steps). The following 4 levels of difficulty were used to evaluate each domain: no difficulty, some difficulty, much difficulty, and unable to perform. An impairment of ADL, IADL, LSA, GPA and LEM was defined if difficulty was reported in the respective domain questions. Participant disability was analyzed as a continuous variable, which was the total number of difficulties within the 5 major domains, with a range from 0 to 19.

### Demographic and disability-related covariates

Demographic information, including sex, age, race, smoking status, and medical history, was collected with computer-assisted personal interviewing methodology, conducted by trained examiners. Besides age was view as continuous variable, other demographic information was view as binary variable. Race/ethnicity was divided into non-Hispanic white and others. Smoking status was classified according to the question “Do you now smoke cigarettes?” Participants were classified as having diabetes mellitus according to the question “Other than during pregnancy, have you ever been told by a doctor or health professional that you have diabetes or sugar diabetes?” or the use of diabetic medication (including insulin injection and/or oral hypoglycemic agent), or a random plasma glucose level of >= 200 mg/dl, or fasting glucose level of >=126 mg/dl. Participants were classified as having hypertension if the mean blood pressure was ≥140/90 mmHg, they were taking anti-hypertensive medication, or had been diagnosed with hypertension by a doctor. Other medical history, such as a stroke, was classified by a question such as “Has a doctor or health professional ever told you that you had a stroke (or coronary heart disease, or angina/angina pectoris)?”

Other disability-related covariates such as BMI, and biochemical analysis results were view as continuous variables. BMI was calculated by dividing the participant’s weight in kilograms by the square of their height in meters. A biochemical analysis was performed through an enzymatic reaction of serum glucose (Cobas Mira assay). Total cholesterol (TC), aspartate aminotransferase (AST) and HDL-C were measured in the Lipoprotein Analytical Laboratory at Johns Hopkins University. All protocols followed the standardized methods with documented accuracy with respect to CDC reference methods. All methods were performed in accordance with the relevant guidelines and regulations of CDC.

### Statistical analyses

All statistical analyses were performed using SPSS (Version 18.0 for Windows, SPSS, Inc., Chicago, IL, USA). Quantitative parameters were indicated as means and standard deviations (SD). Demographic characteristics were compared by an independent t-test; a Wilcoxon rank sum test was used for continuous variables and Chi-square test for categorical variables. Two-sided p-values of less than 0.05 were considered significant. Covariate adjustments were investigated by the following extended-model linear regression: Model 1 was adjusted for age, sex, race/ethnicity and BMI. Model 2 = Model 1 + serum fasting glucose, TC, HDL-C, and AST. Model 3 = Model 2 + history of coronary heart disease, congestive heart failure, stroke, hypertension, diabetes, current smoking status, and experience of memory problem.
